# Ectopic lipid deposition in kidney diseases: mechanisms in specific cell types and therapeutic strategies

**DOI:** 10.3389/fendo.2026.1788998

**Published:** 2026-04-22

**Authors:** Qi Zhong, Yue Zhang, Di Sun, Jin Peng, Peng Zhang, Ruolin Wang, Lei Cao, Hailong Chen, Chunwei Wu, Ze He

**Affiliations:** 1 College of Traditional Chinese Medicine, Changchun University of Chinese Medicine, Changchun, China; 2 Department of Endocrinology and Metabolism, The Affiliated Hospital of Changchun University of Chinese Medicine, Changchun, China

**Keywords:** cell-specific, ectopic lipid deposition, kidney diseases, molecular mechanisms, therapeutic prospects

## Abstract

Kidney diseases represent a major global health burden and arise from complex interactions among multiple cellular and molecular processes. Ectopic lipid deposition (ELD), defined as the accumulation of excess lipids in non-adipose tissues when lipid supply exceeds adipose storage capacity, has emerged as a key contributor to the initiation and progression of renal injury. ELD occurs across multiple renal cell types, including proximal tubular epithelial cells, podocytes, mesangial cells, glomerular endothelial cells, as well as interstitial fibroblasts and macrophages. Owing to differences in metabolic profiles and lipid-handling capacity, these cells exhibit distinct susceptibilities and pathological responses to lipid accumulation. This review summarizes the major sources and underlying mechanisms of renal ELD, with particular emphasis on cell-specific injury pathways driven by different lipid subtypes. It further discusses how these divergent responses collectively contribute to renal dysfunction and structural damage. We also outline current approaches for the clinical assessment and diagnosis of renal ELD, and highlight the relevance of age stratification in improving diagnostic precision. Recent advances in therapeutic strategies targeting renal ELD are also reviewed, including evidence from diabetic kidney disease, obesity-related kidney disease, acute kidney injury, and Alport syndrome. Overall, this review provides a systematic overview of the molecular mechanisms and therapeutic implications of ELD in kidney diseases from a cell-specific perspective, and highlights its potential as a target for improved prevention and treatment strategies.

## 1 Introduction

Kidney diseases have become a major global health threat. According to the Global Kidney Health Alliance, over 800 million people worldwide are affected by kidney diseases (1, 2). The causes of kidney diseases are diverse and multifactorial, often involving complex interactions between genetic, environmental, and metabolic factors, with a lack of effective prevention, early detection, and treatment strategies. Kidney diseases not only decrease patients’ quality of life but also place a significant burden on public health systems. This situation calls for the development of new diagnostic tools and innovative therapeutic approaches (3). Among the various factors contributing to the progression of kidney diseases, lipid metabolism disorders have emerged as critical risk factors. Epidemiological studies have shown that dyslipidemia can impair renal function and accelerate the progression of kidney diseases (4, 5).

Lipids are crucial biomolecules that play a key role in maintaining cell structure and function. These include various types, such as triglycerides, phospholipids, cholesterol, and sphingolipids, which not only form the essential structural framework of cell membranes but also participate in numerous physiological processes, including energy storage, signal transduction, and regulation of cellular homeostasis (6, 7). Under physiological conditions in a healthy state, lipids are primarily stored as triglycerides in the adipocytes of white adipose tissue (8, 9). In addition to adipocytes, non-adipose cells such as hepatocytes, cardiomyocytes, and proximal tubular epithelial cells (PTECs) can also synthesize and temporarily store small amounts of lipids to maintain local energy balance. However, when circulating lipid levels exceed the storage capacity of adipose tissue, excess lipids begin to accumulate in non-adipose tissues, including the kidneys and heart. This process is referred to as ectopic lipid deposition (ELD) (10, 11). ELD not only indicates the limited capacity of adipose tissue for energy storage but also serves as an early marker of systemic metabolic imbalance.

The kidney, as a highly metabolically active organ, is a key site for ELD. Lipids in the kidney are primarily stored as intracellular lipid droplets (LDs) across a range of cell types, including PTECs, podocytes, mesangial cells (MCs), glomerular endothelial cells (GEnCs), as well as interstitial fibroblasts and macrophages (12, 13). Importantly, different cell types respond to lipid accumulation in a cell type–specific manner, due to variations in their physiological functions and metabolic pathways. This suggests that renal ELD not only reflects lipid overload but also contributes to kidney disease progression through cell-specific mechanisms of damage. Advances in imaging techniques, such as proton density fat fraction (PDFF) and magnetic resonance imaging (MRI), now enable non-invasive evaluation of renal lipid content (14). In the 1980s, Moorhead et al. proposed that lipids could directly mediate glomerular and tubulointerstitial damage, accelerating kidney disease progression (15). Recent studies have shown that diabetic patients with microalbuminuria have significantly higher renal lipid content compared to healthy controls (16). In type 2 diabetes, renal lipid accumulation is independently associated with an increased risk of chronic kidney disease (CKD), identifying it as a key risk factor (17). These findings highlight the potential pathological significance of renal ELD in kidney injury. Similar mechanisms linking systemic metabolic disturbances to organ-specific lipid deposition have been demonstrated in the liver, pointing to the presence of an inter-organ metabolic regulatory network (18). However, the sources of ectopic lipids and their pathogenic roles in the kidney remain insufficiently understood, particularly at the level of cell-specific injury. Here, we examine how lipid accumulation drives injury across major renal cell types, including PTECs, podocytes, MCs, GEnCs, as well as interstitial fibroblasts and macrophages. We further summarize current approaches for the clinical assessment and biomarkers of ELD, together with therapeutic strategies targeting this process. This narrative review draws on studies retrieved from PubMed, Web of Science, and Scopus up to January 2026, using keyword combinations related to “ectopic lipid deposition”, “kidney diseases”, “renal cell types”, “clinical assessment” and “therapeutic interventions”. By linking cellular mechanisms with clinical observations, this work outlines a framework that connects mechanistic insights to potential therapeutic applications in diabetic kidney disease (DKD), obesity-related glomerulopathy (ORG), and other conditions associated with lipid deposition in the kidney.

## 2 Sources of ectopic lipids in the kidney

ELD results from the disruption of lipid metabolic balance, which involves two main processes: increased lipid input and decreased lipid clearance. Increased input includes exogenous lipids from the bloodstream and endogenous lipid synthesis within the kidney, while decreased clearance is primarily due to impaired fatty acid oxidation (FAO), leading to disrupted lipid metabolism. This section will provide a detailed overview of the formation of ELD in the kidney.

### 2.1 Exogenous sources in the kidney

Under conditions such as high-fat diets, obesity, and insulin resistance, plasma levels of free fatty acids (FFAs) and triglycerides are significantly elevated (19). These changes cause dysfunction of visceral fat, which increases fat mobilization, releasing large amounts of FFAs and lipoprotein particles into the bloodstream, leading to elevated blood lipid concentrations (20). FFAs and lipid-rich particles circulate into the kidney and are absorbed by PTECs, podocytes, and GEnCs, ultimately causing ELD (21). In addition, during insulin resistance, the liver increases the synthesis and secretion of very-low-density lipoprotein (VLDL), further adding to the renal lipid burden (22).

In addition to systemic lipid sources, the perirenal fat surrounding the kidney plays a significant role in local renal lipid deposition. First, perirenal fat exerts an indirect paracrine effect by secreting active factors (23). These include pro-inflammatory cytokines like TNF-α and IL-6, as well as adipokines such as adiponectin, leptin, and exosomal microRNAs. Together, these factors create a chronic low-grade inflammatory environment in the kidney (24). While these factors are not lipids themselves, they can impair renal cell metabolism by inducing insulin resistance and oxidative stress. This process indirectly inhibits FAO and promotes lipid accumulation, exacerbating kidney injury (25). Second, perirenal fat may directly contribute to renal lipid accumulation. The FFAs it releases are likely transported to the renal parenchyma through the microvascular network shared between the kidney and perirenal fat. Studies have shown that perirenal fat thickness is positively correlated with urinary albumin excretion and circulating FFAs (26). Moreover, circulating FFAs in renal venous blood are significantly higher than in the jugular venous blood, supporting the hypothesis that FFAs released from perirenal fat can directly enter the renal circulation through the local vascular network, thereby creating a lipid-rich environment in the kidney and inducing damage (27). Therefore, renal ectopic lipid deposition results not only from elevated lipid levels in the blood but also from abnormal metabolism in the perirenal fat (28).

Beyond circulating lipid influx and local contributions from perirenal fat, the gut–kidney axis has emerged as an important route of inter-organ regulation in renal ELD (29). Gut microbiota–derived metabolites, including short-chain fatty acids (SCFAs), trimethylamine N-oxide (TMAO), and bile acids, reach the kidney via the circulation and modulate pathways involved in lipid metabolism. During gut dysbiosis, reduced SCFA production disrupts cellular energy homeostasis, leading to impaired FAO and subsequent lipid accumulation (30). In parallel, loss of intestinal barrier integrity allows lipopolysaccharide (LPS) to enter the circulation, triggering inflammatory responses that further promote lipid deposition (31). Importantly, these gut-derived signals do not serve as direct lipid substrates in the kidney. Instead, they reshape the metabolic and inflammatory milieu, thereby influencing renal lipid homeostasis. Through these mechanisms, the gut–kidney axis contributes to both the maintenance and disruption of lipid balance in the kidney.

### 2.2 Endogenous sources in the kidney

#### 2.2.1 Increased lipid uptake

Lipid uptake across cell membranes is a key source of renal ELD, and this process is mediated by specific transport proteins (32). In the kidney, various transport proteins participate in this process, either cooperatively or independently. However, their expression, mechanisms of action, and regulation differ significantly. Therefore, targeting the regulatory mechanisms of specific transport proteins may provide a new strategy for the precise treatment of renal lipotoxicity.

Fatty acid transport protein 2 (FATP2) is highly expressed in PTECs, with greater expression and specificity than FATP1 and FATP4 (33). In contrast to the CD36 receptor, FATP2 not only facilitates fatty acid transport across membranes but also has acyl-CoA synthetase activity, immediately activating the absorbed fatty acids into fatty acyl-CoA. This process captures the fatty acids within the cell, ensuring that the transported FFAs can be utilized for lipid synthesis (34). FATP2 is primarily regulated by lipid-sensitive peroxisome proliferator-activated receptor α (PPARα) and sterol regulatory element-binding proteins (SREBPs), which respond directly to the body’s metabolic state and energy needs (35). Chen et al. (36) demonstrated that in both the unilateral ureteral obstruction model and the TGF-β-induced tubular epithelial cell model, the FATP2 inhibitor, Lipofermata, significantly reduced LD formation. Further studies by Khan (37) showed that FATP2-deficient mice (Slc27a2^-^/^-^) had significantly lower apoptosis rates in PTECs during albumin overload experiments compared to wild-type controls. These findings suggest that FATP2-mediated FFA uptake across membranes is a key step in proximal tubular lipid deposition.

Cluster of differentiation 36 (CD36) is a transmembrane protein that mediates the transport of long-chain fatty acids (LCFAs) (38). In the kidney, CD36 is widely distributed in GEnCs, podocytes, distal tubular epithelial cells, and macrophages (39). CD36 has a more complex function: it facilitates LCFA internalization via high-affinity binding but lacks enzymatic activity. More importantly, CD36, a pattern recognition receptor, can also recognize and internalize modified lipoproteins such as oxidized low-density lipoprotein (oxLDL) (40, 41). CD36 activity is regulated by post-translational modifications, including palmitoylation, glycosylation, and phosphorylation, and is upregulated by transcription factors such as C/EBP and PXR in metabolic disorders like diabetes (42). Studies show that CD36 expression is significantly increased in PCSK9 knockout mice, leading to increased lipid accumulation in renal tubules (43). In addition to CD36, the scavenger receptor C-X-C motif chemokine ligand 16 (CXCL16) can also internalize ox-LDL into podocytes (44, 45), The endosome then fuses with the lysosome, forming an endosome-lysosome complex, which leads to abnormal accumulation of cholesterol esters and triglycerides in the cytoplasm, accompanied by the release of inflammatory factors and enhanced oxidative stress (46, 47).

FABPs are key regulators of intracellular lipid metabolism, involved in the transport, storage, and biological functions of fatty acids (48). These small, water-soluble proteins bind to LCFAs and other bioactive molecules, facilitating their accurate localization within the cell (49). FABPs facilitate the transport of FFAs from the cytoplasm to mitochondria or the endoplasmic reticulum (ER), where they play essential roles in different metabolic processes. FFAs entering the mitochondria undergo β-oxidation to generate adenosine triphosphate (ATP), which provides energy to the cell, while those transported to the ER are converted into triacylglycerol, accumulating on the ER membrane and forming LDs stored in the cytoplasm. In the kidney, FABP1 is predominantly expressed in proximal tubular cells (50). In the pathological state of DKD, significant renal lipid deposition correlates with decreased FABP1 expression (51), leading to dysregulated fatty acid metabolism and exacerbating renal ELD (52, 53).

#### 2.2.2 Increased lipid synthesis

In addition to circulating lipid uptake, endogenous lipid synthesis in the kidney contributes to lipid deposition. This process mainly involves two core metabolic pathways: *de novo* lipogenesis (DNL) and cholesterol synthesis.

DNL is an energy-demanding biosynthetic process. Acetyl-CoA carboxylase (ACC) catalyzes the conversion of Acetyl-CoA to malonyl-CoA, promoting fatty acid synthesis. Fatty acid synthase (FASN) then converts malonyl-CoA into saturated fatty acids such as palmitic acid (54). Studies have shown that proximal tubular-specific knockout of FASN or acetyl-CoA synthetase 2 in mice significantly reduces lipid deposition (55, 56). Stearoyl-CoA desaturase 1 (SCD1) is a lipogenic enzyme that converts saturated fatty acids into monounsaturated ones. In DKD, upregulated SCD1 expression promotes excessive LD formation and exacerbates renal lipotoxicity (57, 58).

It is worth noting that DNL and FAO are closely linked and together regulate lipid metabolism in the kidney. On one hand, malonyl-CoA inhibits carnitine palmitoyltransferase 1 (CPT1), which is located on the mitochondrial membrane, resulting in impaired FAO. On the other hand, Acetyl-CoA produced via β-oxidation is transported back to the cytoplasm through the citrate-pyruvate shuttle system, where it serves as a substrate for DNL, continuing lipid synthesis and further exacerbating lipid accumulation.

Unlike DNL, the cholesterol synthesis pathway is primarily regulated by the SREBP2-SCAP-HMGCR axis. SREBP2, assisted by its partner protein SREBP cleavage-activating protein (SCAP), is transported from the endoplasmic reticulum to the Golgi apparatus, where it undergoes cleavage and activation by a protease. This activation upregulates key enzymes like 3-Hydroxy-3-Methylglutaryl-CoA Reductase (HMGCR), driving cholesterol biosynthesis (59).

Transcriptional regulation of renal DNL and cholesterol synthesis is controlled by the SREBP family and carbohydrate response element-binding protein (ChREBP), both of which respond to distinct upstream signals, creating a precise regulatory system. The SREBP family consists primarily of SREBP-1c, SREBP-2, and SREBP-1a (60). SREBP-1c is mainly activated by insulin signaling, which in turn activates genes involved in fatty acid and triglyceride synthesis (61). SREBP-2 primarily regulates the transcription of genes related to cholesterol biosynthesis, while SREBP-1a can simultaneously regulate both pathways (62). Animal studies have shown that the activation of the SREBP pathway is closely linked to renal lipid deposition. For example, in rat models, enhanced SREBP expression and increased renal lipids occur simultaneously (63). ChREBP, a glucose-responsive transcription factor, is a key mediator between high-glucose environments and renal lipid accumulation. High glucose leads to the accumulation of intracellular metabolites, which directly activate ChREBP, inducing the expression of key DNL genes like ACC and FASN (64). Due to differences in upstream regulation, renal DNL activation varies depending on the context. In DKD, sustained hyperglycemia is the primary driver, strongly activating ChREBP and leading to “glucose-driven” lipid synthesis. In ORG, hyperinsulinemia plays a more critical role by activating SREBP-1c, driving “insulin-driven” lipid synthesis.

In the setting of metabolic dysregulation, the decreased activity of AMPK, coupled with the sustained activation of mTORC1 signaling, leads to an imbalance that drives the upregulation of lipid synthesis pathways. As a cellular energy sensor, AMPK inhibits ACC via phosphorylation, which lowers malonyl-CoA levels. This suppresses DNL while relieving CPT1 inhibition, thus promoting FAO (65). Therefore, the activation of AMPK, through these dual mechanisms, shifts lipid metabolism toward catabolism and plays a critical role in renal lipid metabolism (66, 67).

#### 2.2.3 Fatty acid oxidation dysfunction

FAO is a critical metabolic pathway in the kidney, particularly in proximal tubular cells, which are rich in mitochondria. It is responsible for converting fatty acids into ATP, providing energy for functions such as active tubular reabsorption (68). However, under pathological conditions, FAO dysfunction creates a vicious cycle that drives the ELD, further exacerbating kidney injury.

FAO is the primary pathway for lipid clearance in the kidney, and its dysfunction directly results in lipid accumulation (69). The rate-limiting step in this process is catalyzed by carnitine palmitoyltransferase 1A (CPT1A), which transports long-chain fatty acids into the mitochondrial matrix for β-oxidation. An animal study showed that rhein enhances FAO by restoring CPT1A activity, improving renal function, thus confirming the pivotal role of CPT1A in FAO (70). At the transcriptional level, the regulatory network of peroxisome proliferator-activated receptor (PPAR) and peroxisome proliferator-activated receptor gamma coactivator 1α (PGC-1α) is key to maintaining FAO homeostasis (71). PPAR includes three subtypes: PPAR-α, PPAR-β, and PPAR-γ. Among them, PPAR-α is highly expressed in the kidney and promotes FAO and oxidative phosphorylation to maintain renal energy homeostasis. Studies have shown that downregulation of PPAR-α expression in DKD mouse models leads to FAO dysfunction (72). Herman-Edelstein et al. (51) confirmed through analysis of renal biopsy samples from DKD patients that downregulation of PPAR-α precedes and leads to significant accumulation of LDs in renal tubules.

With the abnormal accumulation of lipids in renal cells, ELD exacerbates FAO dysfunction. Excess fatty acids and their toxic metabolites induce mitochondrial dysfunction, ROS generation, and endoplasmic reticulum stress, further impairing PPARα/PGC-1α activity and suppressing CPT1A expression, leading to a sustained decline in FAO. These stress conditions also activate pro-fibrotic pathways such as STAT6 and TGF-β, inhibiting FAO at the transcriptional level. Studies have shown that in the unilateral ureteral obstruction model, STAT6 activation inhibits PPARα and its downstream FAO genes, thereby exacerbating lipid deposition and fibrosis (73). In contrast, mitochondrial deacetylase Sirtuin 3 (SIRT3) enhances PPARα activity, upregulating FAO-related genes and alleviating renal lipid accumulation (74). FAO dysfunction and ELD create a self-reinforcing vicious cycle. Impaired FAO is the primary cause of abnormal lipid deposition, and the resulting ELD worsens FAO inhibition by inducing oxidative stress and activating inhibitory signaling pathways. This mechanism plays a central role in the progressive worsening of kidney injury.

**Figure 1:**
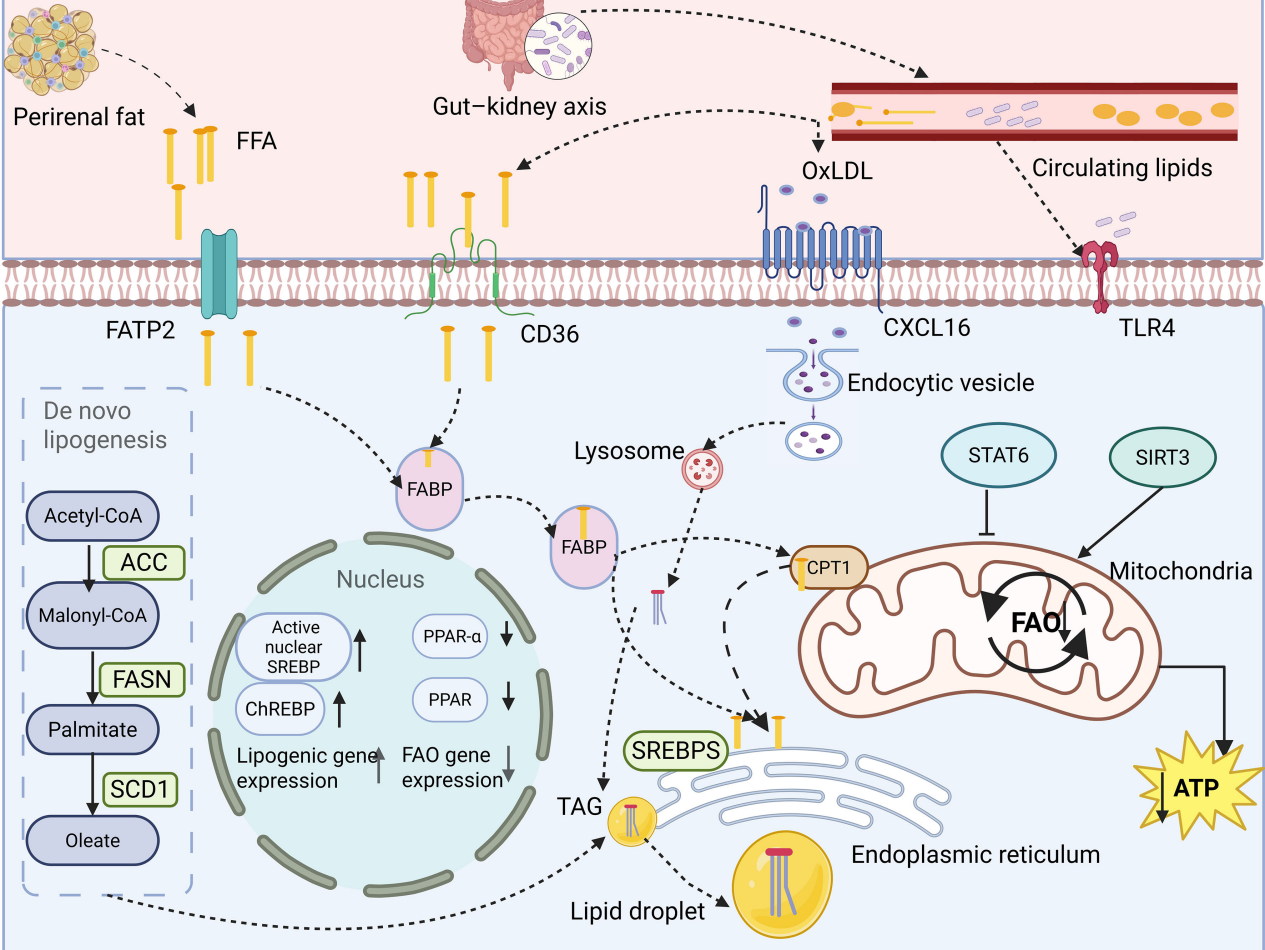
Mechanisms of the origins of ectopic lipid deposition in the kidney. Ectopic lipid deposition in the kidney arises from increased exogenous lipid supply, dysregulated intrarenal lipid metabolism, and cross-organ metabolic regulation. Exogenous lipid sources include lipids from the circulation or adipose tissue, whereas endogenous lipid accumulation results from increased lipid uptake, enhanced lipid synthesis, and impaired fatty acid oxidation.

In summary, ELD in the kidney arises from increased exogenous lipid supply and metabolic imbalance within the kidney. The mechanism of ELD in the kidney is shown in **Figure 1**.

## 3 Cell-specific mechanisms of ectopic lipid deposition-induced renal injury

Lipid overload predominantly occurs in the renal tubules and glomeruli and may also extend to the renal interstitium. In the renal tubules, lipid accumulation mainly occurs in PTECs. In the glomeruli, it involves podocytes, MCs, and GEnCs, whereas in the renal interstitium, it primarily affects fibroblasts and macrophages. Increasing evidence suggests that oxidative stress, inflammation, and organelle dysfunction are key factors in the initiation and progression of renal lipotoxicity, and differences among lipid species in their metabolic characteristics and cytotoxic potential are also increasingly recognized as important contributors to this process.

However, existing literature often focuses on single molecular pathways, assuming that different renal parenchymal cells exhibit similar pathological responses to lipid overload. In reality, kidney cell types differ significantly in energy metabolism, lipid processing, and stress tolerance. In parallel, different lipid subtypes (such as saturated fatty acids, cholesterol and its derivatives, and sphingolipids) exhibit distinct intracellular metabolic fates and toxic effects. The interplay between cellular heterogeneity and lipid diversity may ultimately determine the heterogeneity of ELD-induced injury across different cell types (75). Therefore, ELD may trigger distinct pathological processes in each cell type. This review will systematically explore the key mechanisms of ELD in PTECs, podocytes, MCs, GEnCs, as well as renal interstitial fibroblasts and macrophages, focusing on cell-type specificity.

### 3.1 Proximal tubular epithelial cells

The renal tubules and interstitial tissue make up about 90% of the renal parenchyma, with PTECs being the most abundant and metabolically active cell type in the kidney. PTECs are essential for glomerular filtrate reabsorption. Their physiological activities rely heavily on energy supply, making them particularly vulnerable to metabolic disorders and oxidative stress (76). Increasing evidence shows that in diabetes and related metabolic disorders, excessive lipid deposition in PTECs is a prominent manifestation of ELD in the kidney (77). ELD induces mitochondrial dysfunction, inflammation, oxidative stress, and cell death in PTECs, ultimately leading to structural and functional damage in the proximal tubules and accelerating the decline of kidney function. Moreover, accumulating evidence suggests that different lipid species, such as triglycerides, FFAs, ceramides, and cholesterol esters, contribute differentially to PTEC injury.

Under physiological conditions, PTECs primarily rely on FAO to meet their high energy needs (78, 79). Circulating FFAs continuously enter PTECs through CD36 and FATP2, where they are transported to mitochondria for β-oxidation and oxidative phosphorylation, generating ATP (80). However, in metabolic dysregulation associated with diabetes, increased lipid uptake and decreased FAO capacity coexist, leading to impaired lipid clearance in PTECs. Excessive FFAs gradually accumulate as LDs in the cytoplasm (81–83). Among different fatty acid subtypes, saturated fatty acids (e.g., palmitic acid) and unsaturated fatty acids (e.g., oleic acid) are both taken up via CD36 and FATP2, yet display distinct intracellular fates. Saturated fatty acids readily induce mitochondrial dysfunction and endoplasmic reticulum stress, whereas unsaturated fatty acids are preferentially esterified into triglycerides and sequestered into LDs, thereby exhibiting relatively lower cytotoxicity (84). Notably, unsaturated fatty acids such as oleic acid can also attenuate palmitic acid-induced lipotoxicity, suggesting a protective role under conditions of lipid overload. Additionally, in pathological conditions such as proteinuria, kidney injury molecule-1 (KIM-1) is induced and overexpressed on the apical membrane of PTECs. KIM-1 is a key biomarker for tubular injury and acts as a phagocytic receptor, recognizing and internalizing palmitic acid–albumin complexes. After lysosomal degradation, palmitic acid released from the complex is esterified into neutral lipids like triglycerides, which accumulate in the cytoplasm (85). Animal studies show that deleting or blocking the KIM-1 gene significantly reduces LD accumulation in renal tubules and partially alleviates structural and functional damage, suggesting that KIM-1-mediated lipid uptake plays an important role in renal tubular lipotoxicity (86). From a metabolic regulation perspective, lipid accumulation in PTECs under ELD conditions results not only from increased lipid input but also from impaired FAO regulatory networks. Several studies show that key transcriptional axes regulating FAO, such as PPAR-α/PGC-1α and mitochondrial deacetylase SIRT3, are suppressed under conditions like DKD, limiting fatty acid entry into mitochondria and their involvement in oxidative metabolism (87, 88). This indicates that in ELD, PTECs accumulate lipids not only due to increased lipid input but also because of continuous suppression of the FAO-centered metabolic program, which fosters the persistent accumulation of ectopic lipids.

In a lipotoxic environment, mitochondria, as the central organelles for PTEC energy metabolism, are considered key drivers in the initiation and progression of tubular injury (89, 90). First, the continuous overload of fatty acid substrates inhibits mitochondrial β-oxidation, leading to a decrease in ATP production and excessive generation of mitochondrial reactive oxygen species (mtROS) (91). Among different lipid subtypes, saturated fatty acids are particularly detrimental to mitochondrial function. Palmitic acid promotes the opening of the mitochondrial permeability transition pore, dissipates membrane potential, and drives excessive mtROS generation. Furthermore, ceramide, a representative sphingolipid, exacerbates mitochondrial injury by disrupting cardiolipin organization and impairing the activity of electron transport chain complexes (92). Excessive mtROS not only damage mitochondrial membrane lipids and proteins, disrupt the cristae structure and membrane potential, but also diffuse into the cytoplasm. This triggers lipid peroxidation, protein carbonylation, and DNA damage, resulting in widespread oxidative stress. The interplay between energy metabolism dysfunction and oxidative stress exacerbates both, further worsening mitochondrial dysfunction (93).

At the same time, lipotoxic stress impairs the dynamic balance and quality control mechanisms of mitochondria. mtROS, along with the aberrant activation of related signaling pathways, promotes the activation of the mitochondrial fission protein dynamin-related protein 1 (DRP1), while simultaneously inhibiting the expression and activity of the mitochondrial fusion proteins mitofusin 2 (MFN2) and optic atrophy 1 (OPA1), leading to excessive fragmentation of the mitochondrial network (94, 95). Fragmented mitochondria exhibit reduced cristae structure, unstable membrane potential, decreased oxidative phosphorylation efficiency, and become more susceptible to mitochondrial permeability transition (96).

Furthermore, the PINK1/Parkin-mediated mitophagy process is inhibited, impeding the timely clearance of damaged mitochondria and allowing their continued accumulation within the cell, thereby establishing a vicious cycle (97). Notably, functional interactions between LDs and mitochondria play a crucial role in maintaining the balance of fatty acid supply and oxidation (98). In a lipotoxic state, the disruption of this structure not only hinders the efficient transport of fatty acids into the mitochondria but also promotes the generation of mtROS in the local microenvironment, further aggravating the imbalance in lipid droplet–mitochondrial coupling and energy metabolism (99). Overall, lipotoxicity is not solely a mitochondrial damage event, but a process that, by impairing FAO, disrupting mitochondrial dynamics and quality control, gradually pushes PTECs into a pathological metabolic state of low efficiency and high oxidative stress, leading to the loss of the cell’s ability to compensate for sustained lipid load.

In PTECs, selective autophagy of LDs is a key mechanism for maintaining lipid homeostasis and providing mitochondrial substrates. Under lipotoxic conditions, lipophagy is inhibited, causing lipid droplets to fail to be cleared in time, further exacerbating mitochondrial substrate overload and mtROS generation, forming a vicious cycle of damage (100, 101). LDs enriched with saturated fatty acids are associated with impaired autophagic flux, leading to reduced lipid clearance and sustained lipotoxicity. In contrast, unsaturated fatty acids tend to promote triglyceride-rich lipid droplet formation and are more compatible with autophagy activation. This differential regulation is likely related to alterations in autophagy signaling pathways, lipid metabolism, and lipid droplet-associated proteins, rather than direct differences in autophagic recognition efficiency. Additionally, lipid overload and reactive oxygen species (ROS) can activate pro-inflammatory signaling pathways via NF-κB and NLRP3 inflammasome, increasing the secretion of IL-1β and IL-18, which further harms the tubular structure and adjacent podocytes (102). Studies show that high-fat diet-fed or palmitate-treated mice develop severe inflammation in renal tubular cells, and inhibition of CD36 can reduce the upregulation of NLRP3, Caspase-1, IL-1β, and IL-18, both *in vitro* and *in vivo* (103). Long-term lipotoxicity can also trigger partial epithelial-to-mesenchymal transition (EMT) in PTECs, leading to the release of pro-fibrotic factors like TGF-β and CTGF, resulting in peritubular fibrosis and extracellular matrix (ECM) accumulation (104, 105). Lipotoxicity-induced programmed cell death, including apoptosis, ferroptosis, and necroptosis, accelerates PTEC loss, reducing tubular reabsorption capacity and worsening kidney dysfunction. Different lipid species may differentially contribute to these processes, with saturated fatty acids promoting apoptosis, while ceramides and cholesterol derivatives promote inflammation and fibrotic responses.

In summary, lipid accumulation, mitochondrial damage, and cell death in PTECs under ELD conditions are not isolated events. Rather, they arise from a pathological metabolic remodeling process that begins with FAO downregulation and is continuously amplified by mitochondrial dysfunction and oxidative stress. This process underpins the metabolic vulnerability of PTECs. Considering the distinct effects of specific lipid species adds an additional layer of understanding to PTEC lipotoxicity.

### 3.2 Podocytes

Podocytes are terminally differentiated cells in the kidney. Together with their interdigitating foot processes and slit diaphragms, they form the central structural framework of the glomerular filtration barrier and represent a critical cell type for maintaining filtration selectivity and protein homeostasis (106), podocyte injury ultimately results in proteinuria and progressive disruption of the filtration barrier. In contrast to PTECs, whose energy metabolism relies heavily on mitochondrial FAO, podocytes are characterized by constrained intracellular architecture and relatively limited mitochondrial capacity. Consequently, their energy supply predominantly depends on glycolysis, with oxidative phosphorylation occurring only at low levels and largely confined to the cell body (107). This distinctive metabolic profile suggests that podocytes are not specialized for high metabolic throughput but instead prioritize the long-term preservation of filtration barrier integrity, a feature that may inherently predispose them to injury under conditions of lipid overload.

In metabolic dysregulation states such as diabetes and obesity, elevated circulating FFAs and ox-LDL are persistently taken up by podocytes through lipid transport proteins that are abundantly expressed on their surface. However, in contrast to renal tubular cells, podocytes lack an effective compensatory response to upregulate FAO in the face of increased lipid influx. As a consequence, excess lipids progressively accumulate within the cytoplasm, leading to the formation of LDs (108).

FAO exhibits a high ATP yield relative to oxygen consumption and markedly increases mitochondrial oxygen demand. Under conditions of sustained lipid overload, this metabolic burden predisposes podocytes to a localized mismatch between oxygen supply and demand, thereby creating a state of relative hypoxia (109, 110). In hypoxic settings, hypoxia-inducible factor-1α (HIF-1α) becomes stabilized and translocates to the nucleus, where it promotes a metabolic shift toward glycolysis while concurrently suppressing mitochondrial oxidative metabolism. This reprogramming leads to persistent intracellular lactate accumulation, acidification of the intracellular milieu, and subsequent podocyte injury (111). Previous studies have demonstrated that exposure to high glucose, hypoxia, and lipotoxic stress significantly increases HIF-1α protein expression in cultured podocytes *in vitro*, implicating HIF-1α in the adaptive regulation of podocyte responses to metabolic stress (112, 113). Notably, existing evidence regarding the role of HIF-1α in podocytes is largely derived from whole-kidney analyses and *in vitro* podocyte models, and whether HIF-1α directly drives metabolic remodeling within podocytes *in vivo* remains to be conclusively established.

One of the most critical and pathogenic consequences of lipid overload in podocytes is the disruption of autophagic homeostasis (114). As long-lived, terminally differentiated cells with minimal regenerative capacity, podocytes rely heavily on constitutive autophagy to sustain organelle turnover and preserve cellular homeostasis (115). In pathological conditions marked by an increased lipid burden, lipophagy, which refers to the autophagy-dependent clearance of LDs, plays a crucial role in the fundamental autophagic machinery (116). ELD has been shown to impair podocyte autophagy through sustained activation of the PI3K/Akt–mTORC1 signaling axis. Aberrant mTORC1 activation not only interferes with the initiation of the ULK1 complex, an early and essential step in autophagosome formation (117, 118), but also limits nuclear translocation of transcription factor EB (TFEB) (119), thereby suppressing the transcription of genes involved in lysosomal biogenesis and autophagy. Saturated fatty acids are particularly potent in activating this pathway, thereby inhibiting ULK1 complex initiation and suppressing autophagosome formation (120). In contrast to PTECs, where lipophagy impairment primarily involves alterations in lipid droplet membrane composition, podocytes exhibit a distinct vulnerability through the accumulation of ceramide, a representative sphingolipid. Ceramide disrupts lysosomal membrane integrity and impairs autophagosome–lysosome fusion, further compromising autophagic flux and contributing to podocyte injury (121). Together, these effects constrain autophagy initiation, reduce lysosome availability, and compromise autophagosome–lysosome fusion efficiency, ultimately leading to impaired autophagic flux. Consistently, mice with podocyte-specific deletion of the essential autophagy gene Atg5 develop age-dependent proteinuria and glomerulosclerosis even in the absence of diabetes, underscoring the indispensable role of basal autophagy in maintaining podocyte viability and glomerular integrity (122).

Following autophagy impairment, damaged mitochondria in podocytes fail to be efficiently eliminated, resulting in sustained accumulation of mtROS. This persistent oxidative stress, together with metabolic intermediates generated under intracellular lipid overload, creates a pronounced danger-associated signaling milieu that favors activation of the NLRP3 inflammasome (123, 124). As a central molecular platform enabling podocytes to sense lipid excess, oxidative damage, and organelle dysfunction, NLRP3 inflammasome activation triggers caspase-1 cleavage, promotes the maturation and release of IL-1β and IL-18, and induces pyroptotic cell death via gasdermin D (GSDMD) (125). Inflammasome-driven programmed cell death leads to an irreversible loss of podocytes, thereby undermining the structural and functional integrity of the glomerular filtration barrier.

Concurrently, lipid accumulation and oxidative stress further aggravate protein misfolding by altering the lipid composition of the endoplasmic reticulum membrane and reducing membrane fluidity, thereby promoting ER stress (126, 127). Clinical evidence indicates that podocytes from patients with various primary glomerular diseases exhibit markedly elevated levels of ER stress markers BiP and CHOP, accompanied by ultrastructural features such as ER dilation observed by electron microscopy, implicating ER stress in podocyte dysfunction (128, 129). Under conditions of persistent ER stress, activation of the IRE1α–TRAF2 complex triggers downstream JNK and NF-κB signaling pathways, leading to amplification of inflammatory and apoptotic responses (130). In parallel, sustained activation of the PERK–ATF4 axis induces CHOP expression, enhances pro-apoptotic signaling, and suppresses anti-apoptotic proteins, ultimately resulting in podocyte apoptosis and depletion (131, 132). Consistently, mouse genetic studies demonstrate that podocyte-specific loss of IRE1α causes proteinuria, foot process effacement, glomerular enlargement, and relative podocyte loss, underscoring the essential role of IRE1α as an ER stress sensor in maintaining podocyte homeostasis (133).

Podocytes are uniquely vulnerable to dysregulated sphingolipid metabolism, distinguishing them from PTECs. Ceramide, a central sphingolipid metabolite, accumulates in podocytes under diabetic conditions and induces ROS-mediated mitochondrial damage (134). Interestingly, distinct sphingolipid metabolites exert opposing effects: sphingosine-1-phosphate aggravates kidney injury, whereas ceramide-1-phosphate confers protection in DKD (135). Moreover, gangliosides located within lipid rafts of the slit diaphragm are essential for preserving actin cytoskeleton integrity and preventing proteinuria (136). These findings indicate that podocyte injury reflects the diverse bioactivities of specific sphingolipid species, a complexity that is less evident in PTECs.

In summary, podocytes display a distinct susceptibility to ELD. This process promotes podocyte pyroptosis and apoptosis through inhibition of autophagy, impairment of mitochondrial quality control, and activation of the NLRP3 inflammasome, while endoplasmic reticulum stress and dysregulation of sphingolipid metabolism further exacerbate cellular injury. Collectively, these pathological events result in podocyte loss and filtration barrier dysfunction.

### 3.3 Mesangial cells

MCs are key stromal cells within the glomerulus, located between adjacent capillary loops. Under physiological conditions, MCs contribute to glomerular microenvironmental homeostasis by providing structural support, regulating capillary tone, and facilitating the clearance of deposited macromolecules (137). Upon exposure to a lipotoxic milieu, MCs undergo a functional transition from homeostatic regulators to chronically activated effector cells, characterized by aberrant proliferation, enhanced inflammatory mediator secretion, and excessive extracellular matrix production. These pathological changes drive mesangial matrix expansion and promote the development of glomerulosclerosis, ultimately resulting in disruption of glomerular architecture and deterioration of filtration function (138).

Similar to other renal cell types, MCs are capable of taking up circulating FFAs through constitutive transport pathways. However, the lipid input route that is more distinctive and pathologically relevant in MCs involves the selective recognition and receptor-mediated endocytosis of lipoprotein particles. Previous studies have demonstrated that mesangial cells possess a strong capacity for binding and internalizing low-density lipoproteins, and that increases in intracellular lipid burden largely depend on receptor-mediated lipoprotein uptake (139). Beyond direct cellular uptake, the mesangial matrix itself can function as a local niche for lipoprotein retention and modification, facilitating glycation or oxidation of low-density lipoproteins and thereby generating modified lipoproteins with enhanced biological activity, cytotoxicity, and pro-inflammatory potential (140). Under lipotoxic conditions, persistent influx and uptake of oxLDL drive aberrant lipid accumulation within MCs and promote the formation of mesangial foam cells, a process widely regarded as an early pathological event underlying mesangial matrix expansion and the subsequent development of glomerulosclerosis (141).

Lipid overload can initiate inflammatory responses in MCs. OxLDL and saturated fatty acids activate the NLRP3 inflammasome and NF-κB signaling pathways, thereby promoting the expression of pro-inflammatory mediators (142, 143). Moreover, Lee et al. showed that palmitic acid triggers mesangial cell inflammation through the p38MAPK/p-CREB/COX2 pathway (144). In contrast to podocytes, where inflammasome activation predominantly induces pyroptosis, the inflammatory response in MCs is more characteristic of a pro-inflammatory and chemokine-secreting phenotype. MCs release mediators such as IL-1β and TNF-α, as well as substantial amounts of chemokines including MCP-1 and CCL2, facilitating the recruitment of circulating monocytes and macrophages into the glomerulus (145). These infiltrating immune cells subsequently release signals such as TGF-β, which sustain local inflammatory and pro-fibrotic signaling, serving as an amplifying mechanism of glomerular microenvironmental imbalance (146). Furthermore, oxidized high-density lipoproteins (oxHDL) can potentiate this response via receptors including CD36, enhancing inflammatory mediator expression and promoting cellular injury (147).

Under conditions of sustained lipid and inflammatory stimulation, profibrotic transcriptional programs in MCs are strongly activated. Among these pathways, the TGF-β/Smad signaling axis serves as a central regulator of extracellular matrix synthesis (148). Evidence indicates that oxLDL can directly activate this pathway, upregulate TGF-β1 and its downstream effector PAI-1, and enhance Smad3 nuclear translocation and transcriptional activity (149). Consequently, activated MCs display both increased proliferative capacity and elevated synthesis of matrix components, including type I and type III collagen and fibronectin, while also suppressing matrix metalloproteinase activity via upregulation of PAI-1 and related mechanisms. This combination of effects disrupts extracellular matrix homeostasis, contributing to progressive mesangial matrix expansion and compression of the glomerular capillary loops, which are key events in the pathogenesis of glomerulosclerosis.

In summary, MCs respond to ELD with prominent inflammatory activation and ECM accumulation. Through foam cell formation, inflammatory cytokine production, and activation of pro-fibrotic signaling pathways, these changes drive mesangial matrix expansion and glomerulosclerosis.

### 3.4 Glomerular endothelial cells

GEnCs constitute a critical component of the glomerular filtration barrier and are distinguished by extensively fenestrated architecture, with their luminal surface coated by a glycosaminoglycan-rich glycocalyx (150). This specialized structure enables GEnCs to maintain efficient blood filtration while regulating vascular permeability, local hemodynamics, and the homeostasis of the glomerular microenvironment (151). Direct exposure to circulating blood renders GEnCs highly responsive to metabolic factors, positioning them as primary targets for systemic metabolic disturbances that can compromise glomerular structure and function (152).

ATP-binding cassette transporter A1 (ABCA1) functions as a central cholesterol efflux transporter in GEnCs, mediating the transfer of excess intracellular cholesterol and phospholipids to ApoA-I for high-density lipoprotein formation, thereby preventing abnormal cholesterol accumulation in non-adipose tissues (153). Persistent uptake of circulating lipids by glomerular endothelial cells, together with impaired ABCA1 expression or transport activity, substantially reduces cholesterol efflux, resulting in intracellular cholesterol accumulation within GEnCs (154). Experimental studies in mouse models indicate that upregulation of ABCA1 can mitigate lipid-induced injury in GEnCs (155).

Abnormal lipid accumulation within GEnCs can initiate and amplify oxidative stress. In addition to oxLDL, other lipid species, including saturated fatty acids and cholesterol esters, may contribute to lipid accumulation and dysfunction in GEnCs. OxLDL and excess cholesterol promote ROS production through several mechanisms, including mitochondrial dysfunction, activation of NADPH oxidase (NOX), and disruption of ER homeostasis (156). Oxidative stress can induce uncoupling of endothelial nitric oxide synthase (eNOS), decreasing nitric oxide (NO) bioavailability and increasing superoxide anion generation, further exacerbating oxidative damage. NO deficiency not only impairs the vasoregulatory function of glomerular capillaries but also compromises endothelial barrier integrity (157). Moreover, oxidative stress can damage the endothelial glycocalyx, increasing plasma protein permeability and thereby contributing to albumin leakage and filtration barrier dysfunction. OxLDL and ROS can also upregulate adhesion molecules such as ICAM-1 and VCAM-1, promoting adhesion and infiltration of circulating monocytes and macrophages into the glomerulus, and recruiting additional immune cells via chemokines including MCP-1 and CCL2, establishing a local inflammatory positive feedback loop that further perturbs the glomerular microenvironment (158).

In addition, lipid overload and oxidative stress may activate profibrotic signaling in glomerular endothelial cells. OxLDL has been shown to stimulate GEnCs to upregulate TGF-β1 and fibronectin expression, thereby promoting ECM synthesis and deposition, and to induce endothelial-to-mesenchymal transition (EndMT), resulting in capillary rarefaction and impaired vascular permeability (159). These alterations contribute to glomerular structural remodeling, sclerosis, and filtration dysfunction, representing a central mechanism underlying early glomerular injury in diabetic kidney disease (160). It is noteworthy that GEnC injury is primarily characterized by barrier dysfunction and microenvironmental imbalance, distinguishing it from tubular injury or injury in MCs.

In summary, GEnCs respond to ELD with barrier dysfunction and microenvironmental dysregulation. ELD disrupts ABCA1-mediated cholesterol efflux, induces oxidative stress, and promotes EndMT, leading to capillary rarefaction, increased permeability, and amplification of local inflammation, ultimately accelerating glomerular injury.

### 3.5 Renal interstitial fibroblasts and macrophages

Interstitial fibroblasts and infiltrating macrophages are key cellular targets of ELD and play critical roles in renal inflammation and fibrosis. Fibroblasts are the principal cell type responsible for maintaining extracellular matrix homeostasis in the renal interstitium (161). During ELD, fibroblasts exhibit a dual role, functioning both as targets of lipotoxic injury and as active drivers of interstitial fibrosis. Under pathological conditions such as high glucose and hypoxia, renal tubular epithelial cells undergo lipid metabolic dysregulation and release inflammatory mediators, including IL-18, VEGF, and MCP-1 (CCL2), via paracrine signaling, thereby triggering inflammatory activation in neighboring interstitial fibroblasts (162). In the kidney, CD36 is highly expressed in pericytes, where it mediates the uptake of fatty acids and oxidized lipoproteins, promoting pericyte-to-myofibroblast transition. Moreover, interstitial fibroblasts may also directly accumulate lipids through CD36-mediated uptake, leading to intracellular lipid accumulation and disruption of metabolic homeostasis, which further exacerbates fibrogenesis (163). Fibroblasts, together with macrophages and inflammatory mediators derived from injured tubular epithelial cells, activate the TGF-β/Smad signaling pathway, upregulate α-smooth muscle actin (α-SMA), and enhance the production of extracellular matrix components, including collagen I, collagen III, and fibronectin (164). Consequently, activated fibroblasts become the primary effector cells driving interstitial fibrosis. The excessive extracellular matrix they produce progressively replaces normal interstitial architecture, resulting in tubular atrophy and capillary rarefaction, ultimately accelerating renal function decline.

Macrophages are central components of the immune system and can undergo polarization into pro-inflammatory M1 and anti-inflammatory M2 phenotypes in response to microenvironmental cues (165). In the kidney, macrophages reside in the glomerular mesangial region and can also infiltrate the renal interstitium. Under conditions of lipid overload, infiltrating interstitial macrophages act as key effector cells driving inflammatory responses. Saturated fatty acids and oxLDL represent major lipid species that modulate macrophage function. In a high-glucose environment, saturated fatty acids activate Toll-like receptor 2 and 4 (TLR2 and TLR4)-dependent signaling, triggering pro-inflammatory pathways including IRF3, AP-1, and NF-κB, thereby promoting M1 polarization and enhancing the production of inflammatory mediators (166). In contrast, oxLDL is taken up by macrophages via scavenger receptors, and this process escapes feedback regulation by intracellular cholesterol levels. As a result, excess free cholesterol is esterified and accumulates as LDs, leading to foam cell formation (166). Furthermore, extracellular vesicles released from lipotoxic tubular epithelial cells can induce IL-1β and TNF-α expression and secretion in macrophages, thereby propagating tubular injury signals to interstitial macrophages and establishing a pathogenic tubule–interstitium crosstalk network (167). Enzymes involved in lipid esterification and degradation also play crucial roles in macrophage functional regulation. Deficiency of adipose triglyceride lipase attenuates pro-inflammatory gene expression while promoting anti-inflammatory macrophage activation (168), whereas inhibition of lysosomal acid lipase drives M2 polarization accompanied by reduced mitochondrial oxidative respiration. These findings highlight the essential role of lipid catabolism in determining macrophage polarization states (169). In summary, interstitial fibroblasts and macrophages serve as key cellular mediators of ELD. Fibroblasts undergo lipid uptake and paracrine activation, leading to myofibroblast differentiation and subsequent extracellular matrix deposition and interstitial fibrosis. In parallel, macrophages accumulate lipids, driving polarization toward a pro-inflammatory phenotype or foam cell formation, thereby amplifying local inflammatory responses. These two cell populations function in concert and, through dynamic crosstalk with TECs, collectively constitute a pathogenic network that drives renal interstitial injury.

**Figure 2:**
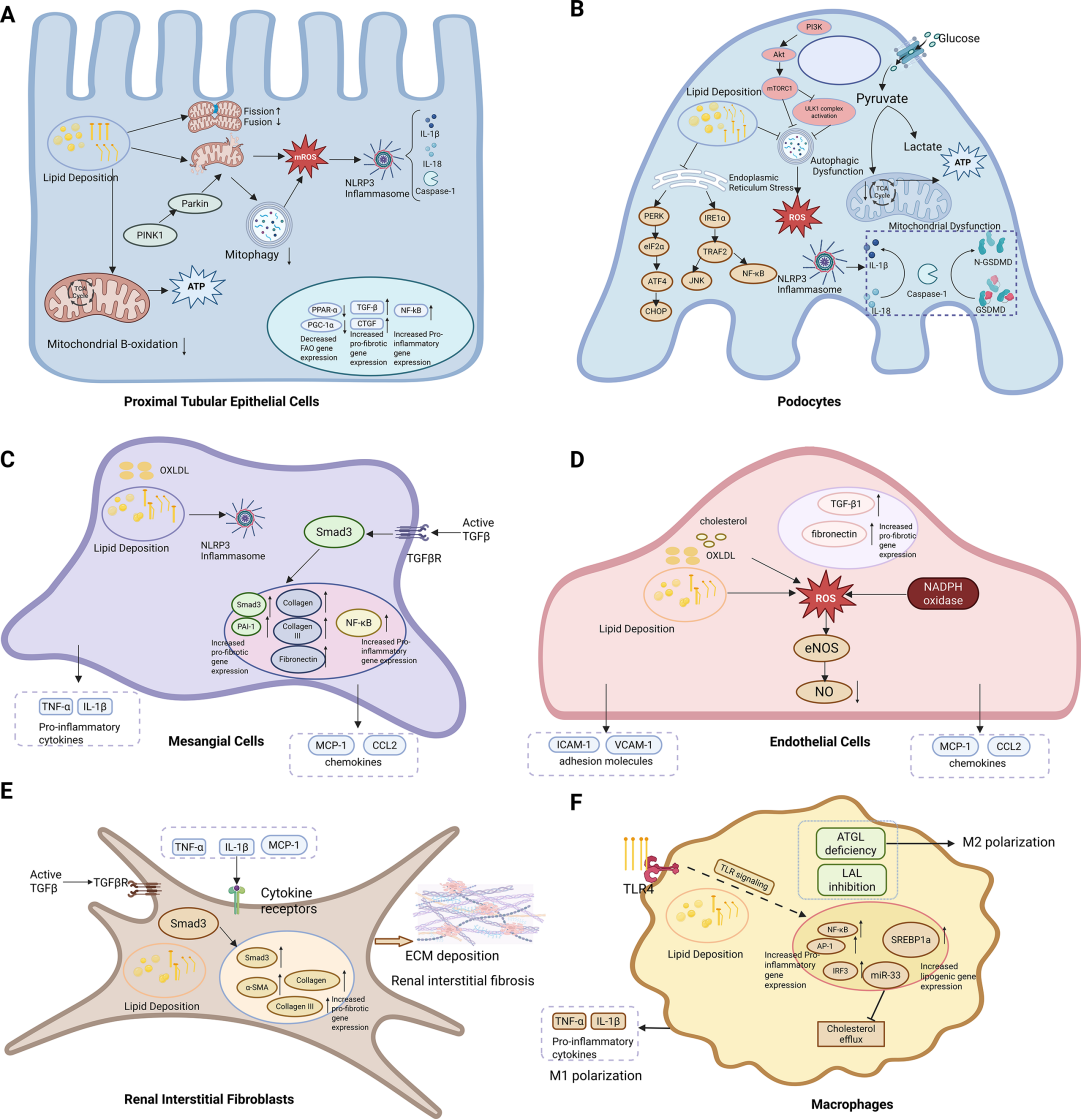
Molecular mechanisms of kidney injury caused by ectopic lipid deposition in cells. **(A)** Proximal Tubular Epithelial Cells: In PTECs, ELD leads to impaired FAO and mitochondrial dysfunction. The accumulation of lipids induces mtROS production, which activates the NLRP3 inflammasome and triggers the release of IL-1β and IL-18. This process also reduces the expression of PPAR-α and PGC-1α, leading to decreased FAO capacity and increased expression of pro-inflammatory genes, further exacerbating kidney damage. **(B)** Podocytes: In podocytes, lipid deposition is exacerbated by glucose metabolism and mitochondrial dysfunction. Imbalanced mitochondrial dynamics, including inhibited fusion and excessive fission, leads to mtROS generation, which activates the NLRP3 inflammasome, inducing inflammation and caspase-1-mediated cell death. At the same time, impaired autophagy worsens lipid accumulation and mitochondrial dysfunction. **(C)** Mesangial Cells: In MCs, ELD and oxLDL activate the NLRP3 inflammasome and trigger the expression of pro-inflammatory cytokines. Activation of the TGF-β/Smad3 pathway promotes mesangial cell proliferation and extracellular matrix synthesis, leading to tubular sclerosis and kidney dysfunction. The accumulation of lipids also increases the production of chemokines, recruiting immune cells and amplifying the inflammatory response. **(D)** Endothelial Cells: In GEnCs, lipid deposition and the accumulation of oxLDL disrupt mitochondrial function and promote ROS production. This activates NADPH oxidase, leading to eNOS uncoupling and reduced bioavailability of NO, impairing vascular function. ROS also upregulate adhesion molecules, increasing immune cell adhesion and infiltration. Additionally, the activation of TGF-β1 and increased expression of fibronectin promote extracellular matrix deposition, leading to endothelial-to-mesenchymal transition, further exacerbating tubular sclerosis and kidney damage. **(E)** Renal interstitial fibroblasts: In renal interstitial fibroblasts, ELD induces fibroblast activation by promoting lipid accumulation and enhancing paracrine signals derived from injured tubular epithelial cells, and activates the TGF-β/Smad3 signaling pathway, thereby promoting the expression of extracellular matrix components such as α-SMA and collagen, ultimately driving myofibroblast differentiation and renal interstitial fibrosis. **(F)** Macrophages: In macrophages, ELD promotes lipid accumulation and drives functional reprogramming. Saturated fatty acids activate the NF-κB, IRF3, and AP-1 pathways through TLR4, promoting M1 polarization and pro-inflammatory gene expression, while ATGL deficiency and LAL inhibition promote M2 polarization, ultimately amplifying inflammatory responses and aggravating renal interstitial injury.

In summary, as shown in **Figure 2**, ELD in the kidney exhibits clear cell-type specificity, with distinct mechanisms of injury across renal cell types.

## 4 Signaling network of renal ectopic lipid deposition and its interaction with systemic metabolic disorders

**Figure 3:**
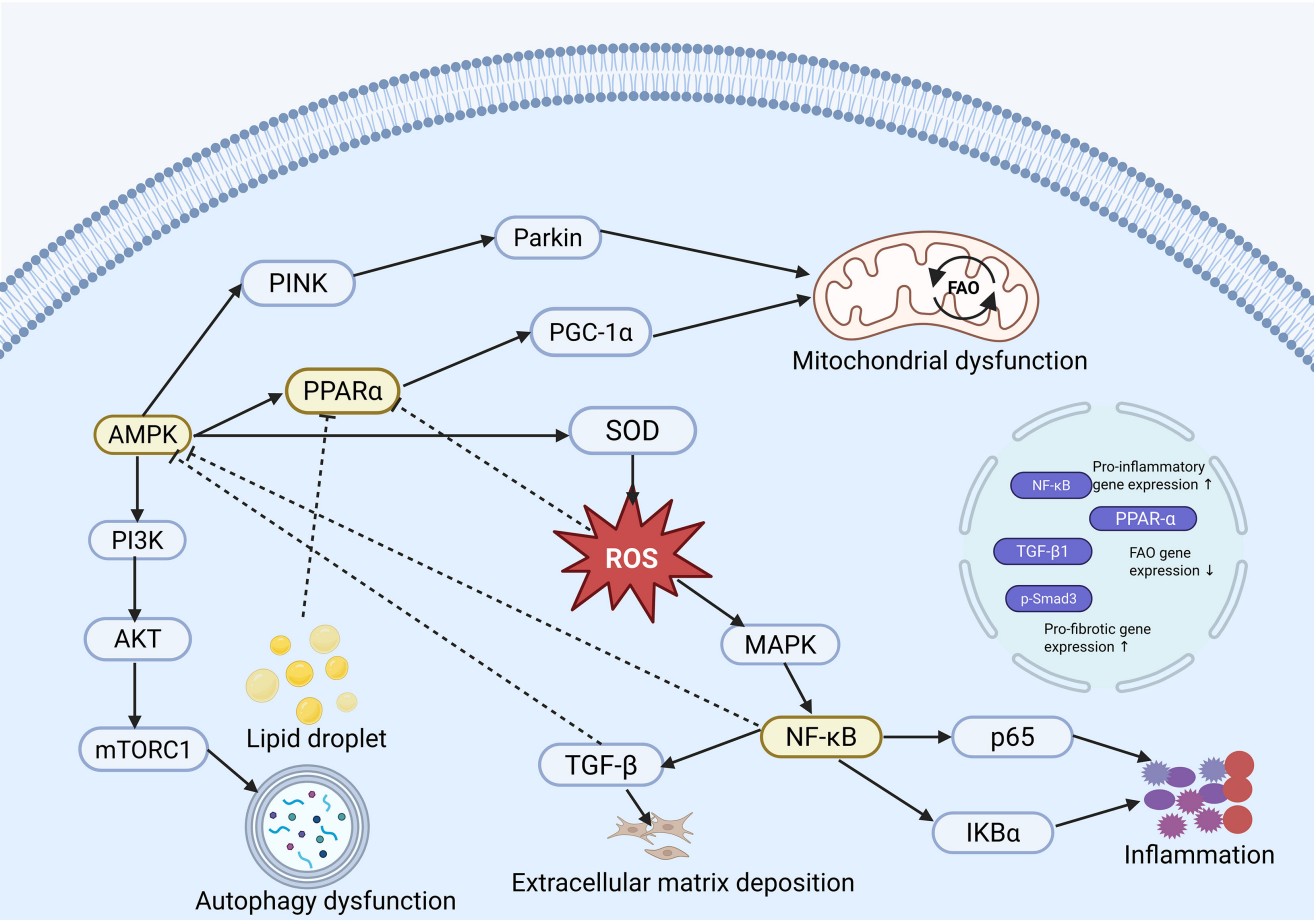
Crosstalk network of renal ELD-associated signaling pathways. AMPK, PPARα, ROS, and NF-κB function as central hubs integrating metabolic, oxidative, and inflammatory signaling in renal ELD. Reduced AMPK activity suppresses PPARα-mediated FAO, promoting lipid accumulation, while ROS generated from lipid overload and mitochondrial dysfunction activates NF-κB and MAPK pathways, driving inflammation. NF-κB further inhibits PPARα, forming a feed-forward loop that links metabolic dysfunction to inflammatory amplification. ROS also reinforces mitochondrial damage, sustaining oxidative stress. TGF-β acts as a downstream effector regulated by ROS and NF-κB, contributing to extracellular matrix deposition and fibrosis. Together, these interactions constitute a self-amplifying, multi-layered regulatory network underlying renal injury progression.

From an integrative perspective, renal ELD-associated signaling does not represent a simple additive process but instead constitutes a dynamic network shaped by extensive crosstalk among multiple pathways. Distinct from the lipid sources and cell-specific injury mechanisms discussed above, this section aims to delineate the interplay among key signaling pathways rather than their isolated effects. Within this network, AMPK, PPARα, ROS, and NF-κB serve as central functional hubs, acting both as convergence points of multiple signaling cascades and as critical nodes linking metabolic dysfunction to tissue injury. The AMPK–PPARα axis and NF-κB signaling form a central interface between metabolic regulation and inflammatory activation. Reduced AMPK activity suppresses PPARα-mediated FAO, thereby promoting lipid accumulation. Concurrently, lipid overload and the resulting ROS activate NF-κB signaling, triggering inflammatory responses. Importantly, NF-κB activation not only represents a downstream consequence of metabolic stress but also feeds back to suppress PPARα transcriptional activity, further impairing FAO and establishing a feed-forward loop between metabolic dysfunction and inflammation (170). ROS functions as a critical integrative node bridging metabolic and inflammatory signaling. On one hand, impaired PPARα activity and mitochondrial dysfunction promote ROS accumulation; on the other hand, ROS amplifies inflammatory signaling through activation of NF-κB and MAPK pathways while further exacerbating mitochondrial damage. These interactions collectively establish a self-reinforcing vicious cycle of metabolic disturbance, oxidative stress, and inflammatory amplification (171). In contrast, TGF-β signaling primarily acts as a downstream effector pathway in fibrosis. Its activation is partially regulated by NF-κB and ROS and contributes to tissue injury by promoting lipid synthesis and extracellular matrix deposition (172). As illustrated in **Figure 3**, renal ELD-associated signaling pathways do not operate in a linear manner but instead form a multi-layered regulatory network centered on key nodes such as AMPK–PPARα, ROS, and NF-κB. Through sustained positive feedback, this network continuously amplifies metabolic dysfunction and drives the progression of renal injury. Such a multi-node, multi-pathway regulatory architecture is highly consistent with systems biology frameworks describing the dynamic evolution of complex diseases (173).

Among specific metabolic disturbances, the interplay between hyperuricemia and renal ELD represents a prototypical bidirectional regulatory relationship. On one hand, elevated uric acid promotes renal lipid accumulation through metabolic dysregulation and inflammatory activation; on the other hand, renal lipid overload impairs uric acid excretion, further exacerbating hyperuricemia. This reciprocal interaction establishes a feed-forward loop characterized by “uric acid elevation–lipid deposition–renal dysfunction”. Collectively, these findings indicate that the relationship between hyperuricemia and renal ELD is not linear but instead constitutes a tightly coupled regulatory network shaped by multi-layered interactions (174).

Similarly, metabolic syndrome is integrated into this regulatory framework through complex metabolic disturbances. Dysfunction of visceral adipose tissue not only enhances lipid influx but also promotes ELD via insulin resistance and chronic low-grade inflammation (175). Concurrently, inflammation triggered by renal lipid overload exerts systemic effects on the liver and adipose tissue through the circulation, further exacerbating systemic metabolic dysfunction and establishing a self-reinforcing inter-organ loop linking adipose tissue, the kidney, and whole-body metabolism (176). Notably, clinical evidence supporting the modulation of systemic metabolic and inflammatory states through targeted interventions has been demonstrated in other diseases, highlighting the broad translational potential of strategies aimed at metabolic network regulation (177).

Therefore, the significance of renal ELD extends beyond its role as a manifestation of local lipid accumulation; it also functions as both an amplifier and a central propagating node within the metabolic network. Through bidirectional cross-organ interactions, renal ELD converts localized metabolic disturbances into systemic metabolic dysfunction and progressive tissue injury.

## 5 Clinical evaluation and diagnosis of renal ELD

As a critical pathophysiological link between metabolic dysfunction and renal injury, the clinical evaluation of renal ELD remains challenging, largely due to the absence of standardized diagnostic criteria and the marked heterogeneity of lipid deposition across nephron segments and patient populations. Current assessment strategies encompass histopathological evaluation, imaging modalities, and liquid biopsy biomarkers, each capturing distinct aspects of renal lipid metabolism, including overall lipid burden, cellular deposition, and dynamic metabolic alterations. Histopathological examination remains the gold standard for ELD assessment. Oil Red O staining of renal biopsy specimens enables direct visualization of intracellular LDs, and studies have shown that individuals with higher BMI are more likely to exhibit focal lipid accumulation within renal cells (178). However, conventional light microscopy lacks sufficient resolution to detect LDs smaller than 0.2–0.5μm. Immunohistochemical detection of the LD-associated protein adipophilin (perilipin-2) overcomes this limitation, allowing for precise identification of small LDs, with signal intensity correlating with intracellular lipid burden and the degree of metabolic dysregulation (179). Imaging modalities hold substantial promise for non-invasive clinical evaluation. Chemical shift-encoded magnetic resonance imaging techniques, such as mDixon-Quant and IDEAL-IQ, enable quantitative assessment of renal fat fraction, providing direct measurement of intrarenal lipid content. Using mDixon-Quant, Yang et al. demonstrated that patients with DKD exhibit significantly higher fat fractions in both the renal cortex and medulla compared with healthy controls and individuals with diabetes alone (180). Furthermore, combined application of three-point Dixon imaging and diffusion tensor imaging has also shown utility in detecting renal ELD (16). Liquid biopsy biomarkers offer a non-invasive and repeatable approach, making them particularly suitable for early detection and monitoring therapeutic responses. Levels of FABP1 and FABP2 increase progressively with the advancement of DKD and serve as indicators of tubular fatty acid load (50). Soluble CD36 (sCD36), the circulating form of the scavenger receptor CD36, has been reported to correlate positively with the urinary albumin-to-creatinine ratio in diabetic kidney disease, and its levels are significantly reduced following treatment with GLP-1 receptor agonists, suggesting its potential as a functional biomarker of renal lipotoxicity (181). In addition, tissue lipidomics enables *in situ* mapping of lipid spatial distribution. High-resolution lipidomic profiling of human kidney tissue has revealed enrichment of specific sphingolipids in glomeruli and associations between distinct phospholipid species and obesity-related renal alterations (182). Despite these advances, a unified diagnostic standard for renal ELD is still lacking. Imaging modalities facilitate non-invasive quantification and longitudinal monitoring, histopathology remains essential for definitive diagnosis, and liquid biopsy biomarkers provide unique advantages for early detection and treatment evaluation. Therefore, a multimodal integrated approach is likely to be critical for improving the accuracy and clinical applicability of ELD diagnosis.

Notably, renal ELD exhibits distinct features across pediatric, adolescent populations and elderly populations, necessitating population-specific diagnostic strategies. In pediatric and adolescent populations, research on renal ELD has primarily been centered on congenital or hereditary kidney diseases rather than primary metabolic disorders (183). For instance, patients with hereditary nephropathies such as Alport syndrome exhibit marked lipid accumulation in renal tissues even in the absence of conventional metabolic risk factors, likely due to gene mutations that disrupt lipid metabolic pathways in podocytes or TECs (184). Moreover, in severely obese adolescents with preserved renal function and no microalbuminuria, urinary sphingolipids—including ceramides, sphingomyelins, and glycosphingolipids—are markedly elevated, indicating early metabolic alterations. Following bariatric surgery, a reduction in body mass index from 50 to 36 kg/m² is accompanied by a significant decrease in urinary sphingolipid levels, suggesting that these lipids may serve as early biomarkers of subclinical renal injury (185). These findings indicate that, in pediatric and adolescent populations, the diagnosis of ELD should place greater emphasis on sensitive liquid biomarkers and metabolic indicators rather than relying solely on conventional measures such as proteinuria or renal function. In contrast, ELD in elderly populations is primarily driven by age-related metabolic remodeling. Aging is associated with structural degeneration and functional decline of the kidney. Chung et al. reported significant downregulation of PPARα expression in aged rat kidneys, accompanied by reduced expression of key FAO enzymes (186). Furthermore, Lee et al. demonstrated that PGC-1α, a critical coactivator of PPARα, is also downregulated in aging kidneys, leading to impaired mitochondrial biogenesis and FAO dysfunction. The coordinated decline of PGC-1α and PPARα imposes a dual constraint on lipid metabolism, rendering intrinsic renal cells more susceptible to metabolic imbalance (187). In addition, elderly individuals commonly present with multiple metabolic comorbidities, polypharmacy, and chronic inflammatory states, which interact with age-related alterations in renal lipid metabolism to exacerbate ELD progression. Therefore, evaluation of ELD in elderly populations should incorporate age-related metabolic disturbances and comorbid conditions, with the establishment of age-stratified diagnostic thresholds and interpretative frameworks, thereby improving diagnostic precision and clinical applicability. Future research should further integrate imaging modalities, molecular biomarkers, and lipidomics data to develop multidimensional diagnostic models, enabling precise stratification and individualized assessment of ELD.

## 6 Intervention strategies targeting ectopic lipid deposition in kidney diseases

ELD is a common metabolic pathological feature across various kidney diseases, fundamentally arising from systemic imbalances in renal lipid uptake, synthesis, oxidation, and efflux. Increasing evidence indicates that, whether in DKD, ORG, or acute and hereditary kidney diseases, ELD continuously drives injury of renal parenchymal cells and disease progression through lipotoxicity, oxidative stress, inflammatory responses, and cell death. Therefore, intervention strategies aimed at renal lipid metabolic pathways, with the alleviation or reversal of ELD as the central goal, are increasingly recognized as a critical component of therapeutic approaches for multiple kidney diseases. Based on this rationale, this review systematically reviews the progress of interventions targeting ELD under different kidney disease contexts, with an emphasis on drugs that have been applied clinically or possess clear translational potential, as well as related interventions derived from natural products, providing references for mechanistic understanding, therapeutic target identification, and optimization and translation of intervention strategies.

### 6.1 Diabetic kidney disease

In recent years, sodium-glucose cotransporter 2 inhibitors (SGLT2 inhibitors) and glucagon-like peptide-1 receptor agonists (GLP-1 receptor agonists) have become key therapeutic options for DKD. SGLT2 inhibitors lower blood glucose by suppressing proximal tubular glucose reabsorption, while GLP-1 receptor agonists promote insulin secretion and reduce appetite, contributing to improved glycemic control. Beyond glycemic regulation, both drug classes have been shown to improve renal function, slow disease progression, and, by modulating renal lipid metabolism, alleviate ELD in DKD, thereby contributing to renal protection.

A recent study demonstrated that the SGLT2 inhibitor Empagliflozin downregulated renal C1QC expression, reducing lipid deposition and inflammation in db/db mice. These effects were recapitulated in high glucose/palmitic acid-stimulated human kidney 2 cells (HK-2), and overexpression of C1QC partially reversed this protective effect (188). In another study, empagliflozin significantly upregulated glomerular ABCA1 expression in db/db mice, promoting cholesterol efflux and inhibiting excessive C3a/C5a activation; this mechanism was further validated in high glucose and high cholesterol-stimulated human renal glomerular endothelial cells (HRGECs) *in vitro* (189). Zhang et al. (190) reported that, in DKD mouse models, Empagliflozin reduced renal tubular lipid accumulation via activation of the AdipoR1/AMPK/p-ACC pathway. The same pathway was demonstrated in high glucose-stimulated HK-2 cells, through which empagliflozin decreased ELD and mitigated cell injury. Furthermore, another SGLT2 inhibitor, dapagliflozin, markedly attenuated podocyte apoptosis and cytoskeletal disorganization in DKD mouse models, while restoring ABCA1 expression and promoting cholesterol efflux. This mechanism was further validated in high glucose-stimulated podocytes via the KLF-5/ABCA1 pathway *in vitro*, alleviating glomerular lipid accumulation and lipotoxicity (191). In addition, Hu et al. found that in both db/db mice and streptozotocin-induced DKD models, Dapagliflozin significantly reduced glomerular triglyceride and FFA accumulation. In high glucose-stimulated podocytes, it promoted FAO via upregulation of ERRα and ACOX1, further alleviating ELD (192).

GLP-1 receptor agonist Liraglutide has been demonstrated to mitigate renal ELD. In a high-fat diet combined with unilateral nephrectomy and STZ-induced DKD rat model, as well as in high glucose-stimulated podocytes *in vitro*, liraglutide alleviated glomerular and podocyte ELD through activation of mTOR/VMP1-mediated autophagy and upregulation of ABCA1, thereby promoting cholesterol efflux (193). In another study focusing on renal tubules, Su et al. reported that Liraglutide effectively reduced LD accumulation in the renal tubules of similar DKD rat models. Mechanistically, in palmitic acid-induced HK-2 cells, liraglutide promoted phosphorylation-mediated activation of AMPK, which in turn upregulated lipolytic enzymes including ATGL and HSL, while inhibiting lipid synthesis proteins such as SREBP-1 and FAS, thereby mitigating lipid deposition (194).

It is important to note that some classic lipid-regulating drugs, such as the PPARα agonist fenofibrate, have mechanisms of action that closely align with renal lipid metabolism regulation. In db/db mice, fenofibrate reduces lipid toxicity by activating the AMPK–PGC-1α–ERRα–p-ACC pathway and inhibiting SREBP-1/ChREBP-1, thereby reducing the accumulation of FFAs and triglycerides in the kidneys. Similarly, in high glucose-stimulated mesangial cells, fenofibrate prevents lipid accumulation-induced cell apoptosis and oxidative stress through the same pathway (195).

In recent years, natural products have attracted considerable attention due to their multi-target effects, low toxicity, and broad pharmacological activity, and their potential to modulate renal ELD has been increasingly recognized in the context of prevalent kidney diseases. The flavonoid quercetin alleviated lipotoxicity in DKD mice by activating the PPARα/PPARγ–UCP1 axis, enhancing FAO, and reducing ROS production; this mechanism was further validated *in vitro* in high glucose-treated HK-2 cells (196). Soyasaponin C, a naturally occurring saponin in soy, upregulated ABCA1 and ATP-binding cassette subfamily G member 1 (ABCG1) via activation of liver X receptor α (LXRα), significantly inhibiting renal cholesterol accumulation and mitigating ELD in db/db mice; similar effects were confirmed in high glucose-treated HK-2 cells (197). Sesamol, a phenolic compound derived from sesame oil, preserved LD–mitochondria contacts and enhanced FAO through activation of the PPARα/PLIN5 pathway, thereby significantly alleviating ELD in DKD mice and *in vitro* in high glucose-treated HK-2 cells; disrupted LD–mitochondria contacts observed in patient renal tissues further supported the clinical relevance of this mechanism (198). Berberine upregulated FAO-related enzymes CPT1, ACOX1, and PPAR-α and activated the AMPK/PGC-1α pathway to enhance FAO in DKD mouse models; this mechanism was further validated in high glucose-treated HK-2 cells (199). Xin et al. (200) further demonstrated, using DKD mice and high glucose-induced podocyte models *in vitro*, that berberine activates the PGC-1α signaling pathway to restore mitochondrial energy homeostasis and FAO in podocytes. Resveratrol, a natural polyphenol, suppressed renal lipid synthesis in DKD mice via inhibition of the JAML/SIRT1 pathway (201). Moreover, the resveratrol derivative pterostilbene inhibited the TGF-β1/Smad3 pathway and downregulated lipid synthesis enzymes including SREBP-1 and FAS, thereby reducing renal lipid synthesis and ameliorating renal fibrosis (202). Collectively, these studies indicate that natural products improve renal lipid metabolism and mitochondrial function through multiple signaling pathways, thereby mitigating renal ELD in DKD. The pharmacological interventions and mechanisms targeting ELD in DKD are shown in **Table 1**.

**Table 1:** Pharmacological interventions and mechanisms targeting ectopic lipid deposition in diabetic kidney disease.

Treatments	*In Vivo*/*In Vitro* models	Targets/signaling pathways	Mechanisms	Reference
Empagliflozin	db/db mice/HK-2	C1QC	Alleviates ELD and inflammation, improving renal tubular function	(188)
Empagliflozin	db/db mice/HRGECs cells	ABCA1	Promotes cholesterol efflux, inhibits C3a/C5a overactivation, alleviates inflammation and renal damage	(189)
Empagliflozin	HFD+ STZ-induced C57 mice/HK-2 cells	AdipoR1/AMPK/p-ACC pathway	Reduces renal tubular ELD and fibrosis, alleviates renal tubular atrophy	(190)
Dapagliflozin	High-fat diet + STZ-induced Wistar rats/podocyte cells	ABCA1, KLF-5	Alleviates podocyte apoptosis, promotes cholesterol efflux, and alleviates glomerular lipid accumulation	(191)
Dapagliflozin	db/db mice/STZ-induced mice/podocyte cells	ERRα, ACOX1	Reduces triglyceride and FFA accumulation, enhances FAO, mitigates lipotoxicity	(192)
Liraglutide	HFD+unilateral nephrectomy +STZ-induced DKD rat model/podocytes	ABCA1, mTOR/VMP1 pathway	Enhances autophagic activity, promotes cholesterol efflux, alleviates glomerular and podocyte lipid deposition and renal injury	(193)
Liraglutide	HFD+unilateral nephrectomy +STZ-induced SD rat model/HK-2 cell	AMPK, SREBP-1, FAS, ATGL, HSL	Activates AMPK phosphorylation, upregulates lipolysis enzymes and inhibits lipid synthesis proteins, alleviating renal tubular lipid deposition	(194)
Fenofibrate	db/db mice/mesangial cells	PPARα, AMPK, PGC-1α, ERRα, p-ACC, SREBP-1, ChREBP-1	Improves lipid metabolism, reduces intra-renal free fatty acid and triglyceride accumulation, alleviates lipotoxicity, and prevents cell apoptosis and oxidative stress	(195)
Quercetin	db/db mice/HK-2 cells	PPARα, PPARγ, UCP1	Enhances fatty acid oxidation, reduces ROS production, and alleviates lipotoxicity	(196)
Soyasaponin C	Soyasaponin C/HK-2 cells	LXRα, ABCA1, ABCG1	Inhibits cholesterol accumulation, alleviates renal fibrosis and inflammation	(197)
Sesamol	HFD+ STZ-induced C57 mice/HK-2 cells	PPARα, PLIN5	Maintain the integrity of the lipid droplet-mitochondria connection, enhances fatty acid oxidation (FAO), and alleviates renal lipotoxicity	(198)
Berberine	db/db mice/HK-2 cells	CPT1, ACOX1, PPAR-α, AMPK/PGC-1α	Upregulates FAO enzymes,enhances FAO, improves mitochondrial function, and alleviates renal tubular ELD	(199)
Berberine	db/db mice/podocytes	PGC-1α	restores mitochondrial energy homeostasis, enhances FAO, alleviates ELD and mitochondrial dysfunction	(200)
Resveratrol	HFD + C57BL/6J mice	JAML/SIRT1	Suppresses lipid synthesis in the kidney, reduces ELD	(201)
Pterostilbene	HFD + C57BL/6J mice	TGF-β1/Smad3, SREBP-1, FAS	Downregulates lipid synthesis enzymes, reduces ELD, alleviates renal tubular damage and fibrosis	(202)

### 6.2 Obesity-related glomerulopathy

ORG is the most common renal complication in obese populations, and its development is closely associated with renal ELD. Consequently, interventions aimed at modulating renal lipid metabolism to alleviate ELD represent a key therapeutic strategy for mitigating ORG (203).

Clinically used hypoglycemic and lipid-lowering drugs, as well as several natural compounds, have demonstrated potential to protect the kidney by improving renal lipid metabolism and alleviating ELD. In terms of clinical drugs, sitagliptin has been shown in ORG rat models to reduce renal ELD. This effect is achieved by downregulating CD36 expression and inhibiting SREBP-1/FAS/ACC-mediated lipid synthesis, while simultaneously upregulating PGC-1α/CPT1 to promote fatty acid β-oxidation (204). Empagliflozin, a SGLT2 inhibitor, decreases FFA uptake in renal tubules and HK-2 cells in high-fat diet-induced mouse models and palmitic acid-treated HK-2 cells by suppressing the PPARγ/CD36 pathway, thereby attenuating ELD (205). Atorvastatin, an HMG-CoA reductase inhibitor, alleviates lipid toxicity resulting from renal lipid accumulation and improves ELD in ORG rat models by inhibiting NF-κB/NOX-4/PKC-α-mediated oxidative stress and inflammation (206). Liraglutide improves renal lipid metabolism and mitochondrial function via the Sirt1/AMPK/PGC-1α pathway in ORG models, thereby reducing ELD and mitigating renal injury (207). Collectively, these findings suggest that targeting renal lipid metabolism to reduce ELD constitutes a crucial mechanism underlying the renal protective effects of these agents.

Regarding natural compounds, Lu et al. (208) reported that the anthocyanin monomer Cyanidin-3-glucoside alleviates renal lipid deposition in ORG mouse models. This effect is mediated by modulation of the gut microbiota and metabolism, as well as inhibition of the proximal tubule PPARγ/CD36 pathway. The mechanism was directly validated in HK-2 cell models, and pathological activation of the same pathway was observed in renal tissues from human ORG patients, providing multi-tiered evidence connecting animal models, cellular mechanisms, and human pathology. Sea cucumber phospholipids, phospholipid compounds extracted from sea cucumbers, were shown in high-fat diet–induced mouse models to reduce renal lipid deposition. This is achieved by downregulating cortical CD36 expression and upregulating PPARα, thereby promoting fatty acid β-oxidation and restoring renal phospholipid homeostasis (209). The natural polysaccharide TPS3A inhibits lipid synthesis and enhances lipolysis in high-fat diet–induced ApoE^-^/^-^ mice by downregulating SREBP-1 and FAS. In palmitic acid-stimulated HK-2 cells, these effects appear to involve the AMPK–SIRT1–FoxO1 signaling pathway (210). Finally, the natural phenolic compound dehydrozingerone coordinates renal lipid synthesis and oxidation in DKD mouse models by activating AMPK signaling, suppressing ACC and SREBP1/2 expression, and upregulating fatty acid oxidation-related proteins PPARα and CPT1. This mechanism was further validated *in vitro* glomerular mesangial cell models (144). The pharmacological interventions and mechanisms targeting ELD in ORG are shown in **Table 2**.

**Table 2:** Pharmacological interventions and mechanisms targeting ectopic lipid deposition in obesity-related glomerulopathy.

Treatments	*In Vivo*/*In Vitro* models	Targets/signaling pathways	Mechanisms	Reference
Sitagliptin	HFD-induced Wistar rats	CD36, SREBP-1/FAS/ACC, PGC-1α/CPT1	Inhibits lipid synthesis, enhances FAO and alleviating renal ELD	(204)
Empagliflozin	HFD induced C57BL/6 mice/HK-2 cell	PPARγ/CD36 pathway	Decreasing FFA uptake in renal tubules and HK-2 cells, alleviating ELD	(205)
Atorvastatin	HFD-induced Wistar rats	NF-κB/NOX-4/PKC-α pathways	reduces oxidative stress and inflammation, alleviates FFA-induced lipotoxicity, and improves renal function by decreasing ELD	(206)
Liraglutide	HFD induced SD rat	Sirt1/AMPK/PGC-1α pathways	Improves renal lipid metabolism and mitochondrial function, reduces ELD, and alleviates renal damage	(207)
Cyanidin-3-glucoside	HFD induced C57BL/6 mice/HK-2 cell	PPARγ/CD36 pathway	C3G alleviates injury in PTCs by improving abnormal glycerophospholipid metabolism and reducing lipid droplet accumulation in PTCs	(208)
Sea Cucumber Phospholipids	HFD induced C57BL/6J mice	CD36, PPARα	Improve renal lipid metabolism, reduce lipid accumulation, and ameliorate renal damage by regulating phospholipid profiles	(209)
Tea Polysaccharides	HFD induced ApoE^-^/^-^ mice/HK-2 cell	SREBP-1, FAS, AMPK, SIRT1, FoxO1	Inhibits lipogenesis and enhances lipolysis, reducing renal tubular ELD	(210)
Dehydrozingerone	HFD induced C57BL/6J mice/mesangial cell	AMPK, ACC, SREBP1/2, PPAR-α, CPT1	Inhibits lipid synthesis, enhances fatty acid oxidation, reduces renal lipid accumulation, and alleviates oxidative stress and inflammation	(144)

### 6.3 Other renal diseases

ELD is widely present in various kidney diseases and constitutes an important common pathogenic mechanism. Acute Kidney Injury (AKI) is not merely a transient structural or hemodynamic abnormality, but a disease state accompanied by significant metabolic reprogramming. Renal lipid metabolic disorder and ELD play a critical role in the occurrence of AKI, impaired repair, and progression to CKD, thus making targeting ELD highly important (211). A recent study demonstrated that the lipid-lowering drug PPARα agonist fenofibrate alleviates ELD and mitigates AKI in mouse models by activating the AMPK signaling pathway, promoting PGC-1α and PPARα expression, and upregulating key FAO enzymes such as CPT1A, CPT2, and ACOX1 (212). The natural flavonoid taxifolin was shown to attenuate renal ELD and improve kidney function in cisplatin-induced AKI mouse models and HK-2 cell models by modulating the FABP4-related lipid metabolism pathway, reversing cisplatin-induced impairment of fatty acid β-oxidation, and upregulating the PGC-1α/PPARα signaling axis (213). Yang et al. (214) validated in AKI mouse models and HK-2 cell models that the biologic agent G9a inhibitor A366 exerts significant renal protective effects by inhibiting the G9a/FXR-CES1 signaling axis, thereby promoting renal FAO and enhancing lipid catabolism. A recent study reported a Klotho gene therapy strategy based on a nano-delivery system, in which nanoparticles capable of specifically recognizing KIM-1 enabled selective delivery of Klotho plasmids to injured TECs; in AKI mouse models, this therapy reduced renal ELD, improved tubular FAO, and alleviated tubular injury and renal interstitial fibrosis by inhibiting p38 and JNK signaling and upregulating PPARα (215). Xu et al. (216) observed in AKI mouse models that the FXR agonist GW4064 upregulates PPARγ expression, thereby enhancing FAO in renal tubular cells; restoration of this metabolic pathway effectively reduces ELD in proximal tubules, mitigates lipotoxicity, and alleviates tubular injury. These findings collectively suggest that restoring renal FAO may serve as a novel therapeutic target for AKI.

With the rising prevalence of metabolic diseases, the incidence of hyperuricemic nephropathy has also been steadily rising. This condition is primarily driven by chronic elevation of serum uric acid, resulting in impaired renal structure and function, and represents a significant form of CKD (217). Recent studies have demonstrated that red ginseng extract, in hyperuricemic mouse models, can downregulate the expression of the lipid synthesis-related protein ACC1 and inhibit inflammatory and fibrotic markers such as TGF-β1 and α-SMA. Through these actions, it modulates renal lipid metabolism, reduces LD accumulation, mitigates inflammation and fibrosis, and alleviates kidney injury induced by hyperuricemia (218).

Notably, in genetic kidney diseases such as Alport syndrome, ELD remains a key mechanism driving disease progression (184). Ge et al. (219) reported in Col4a3^-^/^-^ mice and their primary immortalized podocyte models that empagliflozin induces substrate switching in podocytes by inhibiting pyruvate dehydrogenase (PDH) activity, thereby reducing glucose oxidation, while upregulating CPT1A to enhance FAO. This metabolic reprogramming effectively decreases lipid droplet accumulation in podocytes and mitigates lipotoxicity-induced apoptosis. Ezetimibe, a cholesterol absorption inhibitor used to lower blood cholesterol, was shown in Col4a3^-^/^-^ mice and *in vitro* podocyte models to strengthen lipid droplet–mitochondria coupling, promote fatty acid transport into mitochondria, alleviate renal ELD, and reduce podocyte injury in hereditary glomerular disease (220). The pharmacological interventions and mechanisms targeting ELD in other kidney diseases are shown in **Table 3**.

**Table 3:** Pharmacological interventions and mechanisms targeting ectopic lipid deposition in other kidney disease.

Diseases	Treatments	*In Vivo*/*In Vitro* models	Targets/signaling pathways	Mechanisms	Reference
AKI	Fenofibrate	LPS-induced AKI mice	AMPK, PGC-1α, PPARα, CPT1A, CPT2, ACOX1	Improves FAO by enhancing key enzymes, reduces ELD	(212)
AKI	Taxifolin	Cisplatin-induced AKI mice/HK-2 cell	Fabp4, PGC-1α, PPARα	Restores fatty acid oxidation and alleviates ELD	(213)
AKI	A366 (G9a Inhibitor)	Ischemia-Reperfusion (I/R) injury and cisplatin-induced C57BL/6 mice	G9a, FXR, CES1	Promotes FAO and enhances lipid breakdown	(214)
AKI	Klotho gene therapy	Ischemia-reperfusion injury and folic acid-induced mice	p38, JNK, PPARα	Enhance FAO, reduce renal ELD and alleviate tubular injury and kidney fibrosis	(215)
AKI	FXR agonist GW4064	Cisplatin-induced mice	FXR/PPARγ	Enhances FAO and reduces ELD	(216)
Hyperuricemic nephropathy	Red ginseng extract	HFD induced C57BL/6J mice	ACC1, TGF-β1, α-SMA, OAT1	Improves lipid metabolism, reduces inflammation and fibrosis	(218)
Alport syndrome	Empagliflozin	Col4a3^-^/^-^ mice model/immortalized podocyte model	:::	Induces a metabolic substrate switch in podocytes from glucose to fatty acids, reducing lipid accumulation and apoptosis in podocytes	(219)
Alport syndrome	Ezetimibe	Col4a3^-^/^-^ mice and Col4a3^-^/^-^ podocyte	:::	Enhances lipid droplet-mitochondria coupling, promoting fatty acid transfer, reducing lipid accumulation, and improving mitochondrial function in AS podocytes	(220)

In targeted interventions for ELD, the patient’s metabolic status, baseline renal function, and degree of lipid accumulation may affect drug efficacy, suggesting that individualized medication has potential value. Although dedicated subgroup analyses in ELD remain limited, emerging evidence has begun to inform drug selection across heterogeneous patient populations, and further investigations are warranted to refine intervention strategies and improve clinical decision-making. Building upon individualized therapy, combination strategies have emerged as a promising approach to address the complexity of renal lipid metabolic networks. Such approaches may confer synergistic benefits and enhance overall therapeutic efficacy. Although clinical studies using ELD as a primary endpoint are still lacking, large-scale randomized controlled trials and real-world evidence indicate that combined therapy with SGLT2 inhibitors and GLP-1 receptor agonists can reduce the risk of chronic kidney disease progression, with an acceptable safety profile (221, 222). Mechanistically, the synergistic effects of these combination regimens are well supported. SGLT2 inhibitors promote FAO through AMPK activation, whereas GLP-1 receptor agonists exert both anti-inflammatory and pro-autophagic effects. Together, they exert complementary regulatory actions on lipid metabolism. From a translational perspective, combination therapy may also allow for dose reduction of individual agents while maintaining efficacy, thereby minimizing dose-dependent adverse effects. Furthermore, given the heterogeneity in lipid sensitivity and metabolic demands among different renal cell types, as well as variability in patient-specific pathological contexts, combination therapy offers greater flexibility for individualized intervention. This approach may be particularly beneficial for high-risk populations, including those with multiple metabolic comorbidities, suboptimal response to monotherapy, or rapidly declining renal function.

## 7 Conclusions

Renal ELD arises from the interplay between systemic lipid metabolic imbalance and disruption of intrarenal lipid homeostasis. Accumulating evidence indicates that ELD is not a homogeneous pathological entity but rather a cell type-specific process, in which distinct lipid species exert differential pathogenic effects. Saturated fatty acids (e.g., palmitic acid) primarily induce mitochondrial dysfunction and ER stress, leading to injury in PTECs and podocytes. In contrast, unsaturated fatty acids (e.g., oleic acid) exhibit lower cytotoxicity but can promote LD accumulation under conditions of chronic exposure. Sphingolipids (e.g., ceramides) contribute to inflammatory amplification by disrupting lysosomal membrane integrity in podocytes and activating NF-κB signaling in MCs. Meanwhile, oxLDL, through scavenger receptor-mediated uptake, drives foam cell formation and inflammatory activation in MCs and macrophages. Across renal cell types, ELD triggers a spectrum of metabolic and stress responses in a highly cell type-specific manner. In energy-demanding PTECs, ELD induces metabolic collapse primarily driven by mitochondrial dysfunction. In terminally differentiated podocytes, ELD preferentially disrupts autophagic homeostasis required for maintaining structural integrity. In MCs, ELD drives excessive extracellular matrix deposition and glomerulosclerosis through inflammatory amplification and fibrotic activation. In GEnCs, which serve as the primary interface between the circulation and renal parenchyma, ELD impairs metabolic defense mechanisms, leading to endothelial permeability dysregulation and amplification of local inflammation. In addition, interstitial fibroblasts and macrophages represent key targets of ELD, contributing to interstitial injury through cellular activation and inflammatory amplification, respectively. Collectively, these cell type-specific responses converge to drive injury across renal tubules, glomeruli, and interstitium, highlighting the intrinsic heterogeneity of renal lipid metabolism. Meanwhile, systemic metabolic disorders such as hyperuricemia and metabolic syndrome engage in bidirectional crosstalk with intrarenal ELD by modulating key processes including lipid uptake, synthesis, and oxidation, thereby collectively driving the progression of kidney disease. Collectively, these insights reposition ELD from a localized pathological event to a central hub linking metabolic regulation with tissue injury through cross-organ interactions.

From a clinical perspective, histopathology remains the gold standard for the diagnosis of ELD, whereas imaging modalities and liquid biopsy biomarkers provide emerging tools for non-invasive assessment and dynamic monitoring. Distinct features of renal ELD across different age groups necessitate the establishment of age-stratified diagnostic thresholds to improve diagnostic precision.

Targeting ELD holds significant therapeutic potential in kidney diseases. Emerging evidence suggests that interventions aimed at ELD not only confer benefits in metabolic kidney diseases, such as DKD and ORG, but may also extend to acute kidney injury and hereditary nephropathies, including Alport syndrome. The renoprotective effects of commonly used agents, including SGLT2 inhibitors, GLP-1 receptor agonists, and PPARα agonists, are, at least in part, mediated by their direct modulation of renal ELD, rather than solely their glucose-lowering or lipid-lowering effects. Combination therapy, by targeting complementary pathways, enables synergistic effects and may be particularly advantageous for high-risk populations characterized by multiple metabolic disturbances, suboptimal response to monotherapy, or rapid decline in renal function. In addition, natural bioactive compounds as well as herbal or multi-component formulations regulate renal lipid metabolism through multi-target and multi-pathway mechanisms, representing promising therapeutic avenues for targeting ELD and improving renal outcomes.

Despite substantial advances in recent years, several critical limitations continue to hinder the clinical translation of renal ELD. Most mechanistic studies have treated ELD as a downstream consequence of lipid metabolic imbalance, with limited attention paid to the functional heterogeneity of lipid species, their subcellular localization, and their dynamic remodeling during disease progression, thus oversimplifying the inherent complexity of ELD. Furthermore, current therapeutic strategies have largely focused on reducing total renal lipid burden. However, lipids function not only as lipotoxic stressors but also as essential substrates for maintaining cellular energy homeostasis across different renal cell types. Insufficient understanding of cell type-specific metabolic demands and lipid tolerance thresholds may therefore limit the long-term safety and efficacy of ELD-targeted therapies. At present, most evidence regarding the mechanisms and therapeutic interventions of renal ELD is derived from animal models or *in vitro* systems. Direct evidence from human kidney tissues remains limited, particularly for studies that track dynamic pathological changes during disease progression. Key mechanisms highlighted in this review, including impaired FAO, NLRP3 inflammasome activation, mitochondrial dynamics dysregulation (e.g., Drp1-mediated mitochondrial fragmentation), and endothelial-to-mesenchymal transition, are predominantly supported by findings from rodent models and immortalized cell lines. Their relevance and quantitative contribution to human renal pathophysiology therefore require further validation using human-derived samples and well-designed clinical studies.

Although numerous pharmacological agents have shown promising efficacy in reducing ELD in preclinical studies, their translatability to complex human disease contexts, optimal dosing strategies, long-term efficacy, and potential adverse effects require rigorous validation in large-scale clinical trials. Notably, two critical gaps remain in the current clinical evidence base for targeting ELD. First, although large cohort studies and randomized controlled trials have consistently demonstrated the renoprotective benefits of agents such as SGLT2 inhibitors and GLP-1 receptor agonists, these outcomes are primarily based on composite renal endpoints, including end-stage renal disease, doubling of serum creatinine, and cardiovascular mortality, rather than direct assessment of intrarenal lipid content or lipid-related biomarkers. Whether these benefits are primarily mediated through improvements in ELD, and how treatment responses vary across patient subgroups defined by eGFR stage, age, or proteinuria level, remain largely unresolved. Second, although preclinical studies support the synergistic potential of combination therapy, clinical evidence demonstrating its efficacy in modulating ELD remains scarce. Overall, a substantial translational gap persists between mechanistic insights and clinical application. This underscores the urgent need for experimental models that more accurately recapitulate human renal pathology, as well as for well-designed clinical studies that directly evaluate lipid-related endpoints, to facilitate the translation of ELD-targeted strategies into clinical practice.

Therefore, future research should prioritize the establishment of advanced experimental platforms that more faithfully recapitulate human renal pathology, including human-derived kidney organoids, long-term dynamic multicellular models, and animal models that accurately mimic metabolic abnormalities. Such systems will enable systematic interrogation of the dynamic evolution of ELD across distinct renal cell types and its causal links to cellular dysfunction, thereby bridging the gap between mechanistic insights and clinical pathology. In addition, there remains an urgent need to develop non-invasive biomarkers and imaging approaches for longitudinal monitoring of renal ELD. These tools have the potential to reduce dependence on invasive renal biopsy, facilitate large-scale clinical investigations, enable longitudinal assessment of therapeutic interventions, and allow direct evaluation of treatment efficacy and safety in patients. From a clinical translation perspective, future studies should incorporate age-stratified cohort designs to systematically compare the molecular mechanisms, pathological features, and therapeutic responses of ELD across pediatric, adult, and elderly populations. Such efforts will support the development of individualized treatment strategies based on age, renal function stage, and metabolic status. By identifying differential treatment responses among patient subgroups, this approach will facilitate the transition from uniform treatment paradigms to precision-based interventions. Finally, future therapeutic strategies should shift from broad metabolic modulation toward precise targeting of pathogenic cell populations and key lipid metabolic pathways. Selective regulation of lipid transport, LDs turnover, and FAO within specific renal cell types, combined with stage-specific or combinatorial interventions, may enable precise control of renal lipid metabolism. Such approaches hold the potential to enhance therapeutic efficacy while minimizing risks associated with metabolic compensation and energy imbalance.
